# The dimerization domain of HIV-1 viral infectivity factor Vif is required to block virion incorporation of APOBEC3G

**DOI:** 10.1186/1742-4690-4-81

**Published:** 2007-11-24

**Authors:** James H Miller, Vlad Presnyak, Harold C Smith

**Affiliations:** 1 OyaGen, Inc, 601 Elmwood Ave., Rochester, NY 14642, USA; 2 Department of Biochemistry and Biophysics, 601 Elmwood Ave, Rochester, NY 14642, USA

## Abstract

**Background:**

The HIV-1 accessory protein known as viral infectivity factor or Vif binds to the host defence factor human APOBEC3G (hA3G) and prevents its assembly with viral particles and mediates its elimination through ubiquitination and degradation by the proteosomal pathway. In the absence of Vif, hA3G becomes incorporated within viral particles. During the post entry phase of infection, hA3G attenuates viral replication by binding to the viral RNA genome and deaminating deoxycytidines to form deoxyuridines within single stranded DNA regions of the replicated viral genome. Vif dimerization has been reported to be essential for viral infectivity but the mechanistic requirement for Vif multimerization is unknown.

**Results:**

We demonstrate that a peptide antagonist of Vif dimerization fused to the cell transduction domain of HIV TAT suppresses live HIV-1 infectivity. We show rapid cellular uptake of the peptide and cytoplasmic distribution. Robust suppression of viral infectivity was dependent on the expression of Vif and hA3G. Disruption of Vif multimerization resulted in the production of virions with markedly increased hA3G content and reduced infectivity.

**Conclusion:**

The role of Vif multimerization in viral infectivity of nonpermissive cells has been validated with an antagonist of Vif dimerization. An important part of the mechanism for this antiretroviral effect is that blocking Vif dimerization enables hA3G incorporation within virions. We propose that Vif multimers are required to interact with hA3G to exclude it from viral particles during their assembly. Blocking Vif dimerization is an effective means of sustaining hA3G antiretroviral activity in HIV-1 infected cells. Vif dimerization is therefore a validated target for therapeutic HIV-1/AIDS drug development.

## Background

HIV-1 viral infectivity factor (Vif) is an accessory protein required for productive infection in nonpermissive cells [1-3]. An important mechanism of Vif involves its ability to bind to both Elongin B/C complex of the ubiquitination machinery and to the human host defence factor APOBEC3G (hA3G). Formation of these complexes mediates ubiquitination of hA3G and targets hA3G for destruction by the proteosome [4-11]. In the absence of Vif, hA3G assembles within viral particles [6,12-18] and upon post entry, attenuates viral replication through its interaction with the viral RNA genome [12,19-21]. hA3G also catalyzes dC to dU hypermutation during replication on single stranded proviral DNA, resulting in templating of dG to dA mutations during replication of the coding strand [15,22-28].

Vif homodimerization has been shown to be important for HIV-1 infectivity and to involve amino acids 161PPLP164 [29,30]. Recent chemical cross-linking of Vif *in vitro *suggested Vif forms dimers, trimers and tetramers [31]. The multimerization domain is located C-terminal to the putative SOCS box homology domain (144SLQYLAL150), predicted to be required for Vif interaction with the Elongin B/C complex [7]. A3G binding has been mapped to the N-terminal region of Vif [4,10,32,33].

Mass spectrophotometric analysis of peptides released by proteolysis of chemically cross-linked Vif suggested that there were more intra- and intermolecular contacts involving the N-terminal half of Vif compared to the C-terminal half, suggesting that the N-terminus of Vif may be more ordered [31]. The significance of these findings is unclear in the absence of a crystal structure of Vif and Vif multimers.

Two laboratories have predicted a structure of Vif through computational methods involving comparative modelling of Vif relative to known structural folds in the Protein Database [34,35]. Although the groups used different clades of HIV-1 Vif for modelling, the amino acid sequence immediately flanking and including the dimerization domain (KPPLPSV) and PPLP alone had a similar predicted structure (root mean square deviation of 2.91 Å and 2.49 Å, respectively; personal communication, David H. Mathews). Both models predicted that the dimerization domain lies on the surface of Vif monomers where it would be exposed to solvent and accessible for interacting with other Vif molecules or other proteins.

Using the putative Vif SOCS box and the known crystal structures of other SOCS box proteins, the model of Lv *et al*., also predicted the structure of the heterotrimeric complex of Vif with Elongin B and C. In this model, Vif PPLP remained solvent exposed. Modelling could not predict the structure of Vif dimers and therefore the conformation of PPLP in the interface of Vif dimers is unknown. This underscores the importance of empirically determining whether PPLP is accessible for therapeutic targeting in an infected cell.

Peptide mimics of the dimerization domain have been identified through selection of peptide sequences that bind to Vif using phage display technology [29,30]. These peptides disrupted Vif multimerization *in vitro *as evidenced by co-immunoprecipitation analysis of Vif with different epitope tags. When the peptides were fused to the antenipedia cell transduction sequence and added to cell culture media, they markedly suppressed viral infectivity in nonpermissive cells. These intriguing finds have not been independently confirmed.

In this report two commercial laboratories (ImQuest BioSciences and OyaGen, Inc.) have confirmed that the peptide sequence original identified by Yang *et al*. [29] has anti-viral activity. We show that an eleven amino acid Vif dimerization antagonist peptide derived from the sequence originally reported by Yang *et al*., when fused to the HIV TAT transduction peptide rapidly entered cells and distributed within the cell cytoplasm. This peptide suppressed live HIV-1 viral infectivity in a spreading infection assay. Targeting Vif dimerization resulted in a marked increase in hA3G recovery in viral particles released from cells within 24 hours post-infection and these particles had reduced infectivity. The data demonstrate that Vif dimerization plays an essential role in regulating hA3G and validate the multimerization domain of Vif as a potential drug target for anti-retroviral therapeutic development.

## Results and Discussion

### Vif Dimerization Antagonist Peptide Suppresses HIV-1 Infectivity

HIV-1 requires Vif for productive infectivity of T-lymphocytes, macrophages and dendritic cells expressing hA3G [36-39]. In the absence of Vif, hA3G binds to Gag and viral RNA to become incorporated into viral particles [18,40-42]. The interaction of Vif with hA3G is broadly considered to hold potential for the development of a novel class of antiretroviral therapeutics [25,36,37,43,44].

The Vif dimerization antagonist peptide that was originally reported to suppress viral infectivity [29] consisted of an N-terminal antenipedia homeodomain cell transduction peptide (RQIKIWFQNRRMKWKK) fused to a phage display-selected peptide (SNQGGSPLPRSV). We replaced the insect transduction domain with the HIV TAT transduction domain (YGRKKRRQRRRG) in the synthesis of Peptide 1 (YGRKKRRQRRRGSNQGGSPLPRSV).

**Figure 1:**
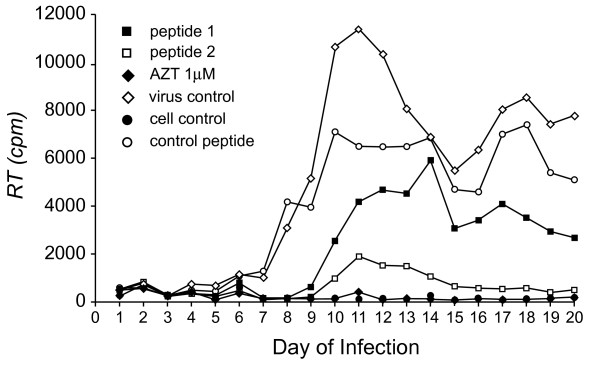
**Vif Dimerization Antagonist Peptides Suppress HIV-1 Infectivity**. MT2 cells grown in microtiter dishes where infected with live HIV-1 virus at 0.01 and treated every other day with either AZT (1 μM), Control peptide (50 μM), Peptide 1 (50 μM) or Peptide 2 (50 μM) or left untreated (viral control) as described in Methods. At the indicated days post-infection, cells were harvested for cell lysate preparation and reverse transcriptase quantification as described in Methods. Lysates were prepared from parallel cultures of uninfected and untreated cells (cell control) as controls for the reverse transcriptase assays.

At ImQuest BioSciences, Peptide 1 was added (final concentration of 50 μM) every other day to the media of cultures of the MT2 nonpermissive cell line that had been infected with live HIV-1_NL4-3 _at moi. of 0.01 to determine its efficacy as an antiviral agent in a spreading infection assay. Viral replication was determined by assaying reverse transcriptase activity in cell lysates. As a control for the effect of cell transduction and the introduction of protein into cells on viral infectivity, a segment of human serum albumin (37DLGEQHFKGLVL48) with an N-terminal TAT sequence was transduced into cells (control peptide). Consistent with previous findings, Peptide 1 reduced viral infectivity relative to the control peptide (Figure 1). This was particularly apparent within the first 9 days of infection and by the end of the study at 20 days. Peptide 1 was not as effective as AZT (1 μM final concentration) in suppressing viral infectivity. Suppression of viral infectivity by Peptide 1 also was observed at higher moi (0.1) and in spreading infections using H9 cells (data not shown).

**Figure 2:**
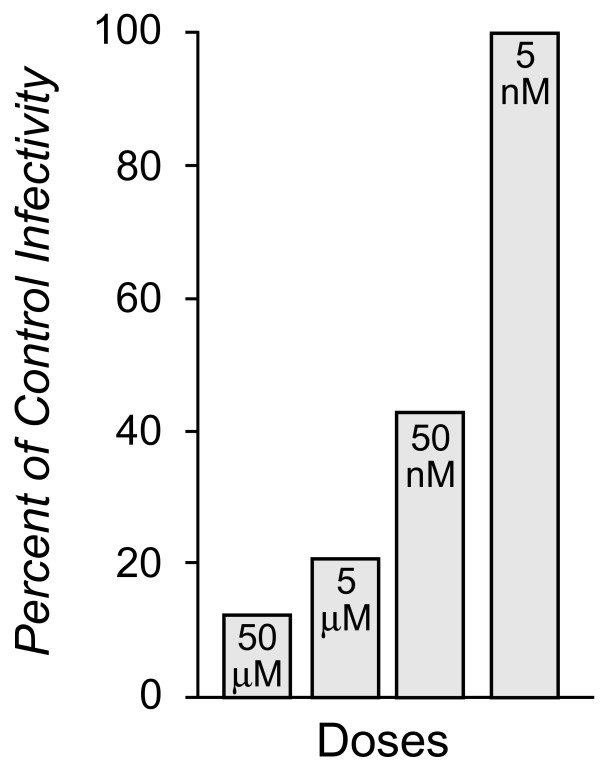
**Peptide 2 has an IC50 of 50 nM**. MT2 cells were treated by Imquest BioSciences with varying doses of Peptide 2 in a spreading infection and infectivity assayed as described in Methods. The percent inhibition of viral infectivity by the peptide was determined during the first seven days of the spreading infection assay. The Inhibitor Concentration, IC (indicated within each histogram) was calculated relative to the untreated virus control.

Peptide 1 does not contain the native Vif amino acid sequence of the dimerization domain (154KPKQIKPPLPRSV167) and therefore we asked what is the minimal sequence of Peptide 1 that would be necessary and sufficient to reduce viral infectivity. Peptide 2 (YGRKKRRQRRRGQGGSPLPSRV) was the shortest peptide synthesized that retained antiviral activity (peptide length requirements determined with live virus in H9 cells through contracted research in the laboratory of Dr. Hui Zhang, Thomas Jefferson University). On a molar basis, Peptide 2 had greater efficacy in suppressing HIV-1 infectivity than Peptide 1 (Figure 1). Analysis of the dose response of viral infectivity to Peptide 2 demonstrated an apparent IC50 of 50 nM. However only an IC85 could be achieved with a dose of 50 μM (Figure 2). Higher doses were not tested. Peptide 2 therefore also is not as effective in inhibiting HIV-1 infectivity as AZT (IC50 of 2–30 nM and IC95 of 5 μM [45,46]).

Peptides with fewer amino acids on the N-terminus or C-terminus of the phage display selected peptide sequence had very low or no ability to suppress viral infectivity (data not shown). All subsequent analyses were conducted with Peptide 2 at 50 uM.

### Validation of the Intracellular Target for Peptide 2

**Figure 3:**
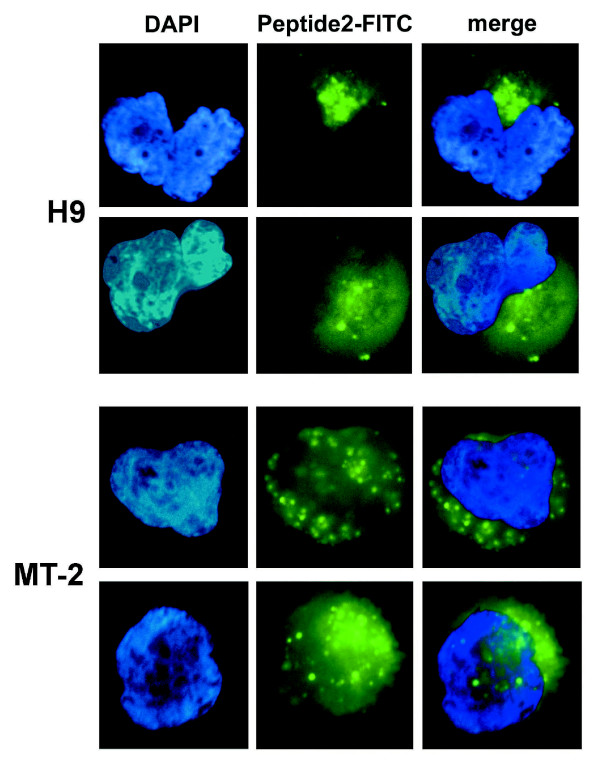
**Intracellular Distribution of Peptide 2**. H9 and MT2 cells were treated with 50 μM Peptide 2-FITC and after 5 minutes of incubation, the cells were fixed, mounted with DAPI-containing media and fluorescence microscopy was performed with filters for DAPI and FITC as described in Methods. Longer durations of treatment were also evaluated in a similar manner. The images were manually overlaid to superimpose the image of the nucleus with each image of Peptide 2-FITC distribution in the cell. Cells from two different regions of the H9 and MT2 plates are shown.

Vif is predominantly a cytoplasmic protein [47,48]. To validate that Peptide 2 entered the cell and thereby had access to Vif, it was synthesized with a C-terminal FITC tag and added to the media of either MT2 or H9 cell cultures. At OyaGen, Inc. cells were fixed at various times after Peptide 2-FITC was added, then washed extensively with phosphate buffered saline and stained with DAPI prior to microscopy to visualize the nuclei of the cells. Fluorescence microscopy revealed an intracellular distribution of Peptide 2-FITC within 5 minutes of its addition to the cell culture media (Figure 3). There was no evidence for plasma membrane accumulation. Peptide 2-FITC localization was predominantly cytoplasmic and remained so for up to 24 hours, diminishing in fluorescence intensity over time (t_1/2 _= 7 to 12 h).

The cytoplasmic localized peptide appeared both punctate and diffuse (Figure 3), consistent with an initial pinocytotic uptake of the peptide followed by TAT-mediated intracellular diffusion [49]. A similar distribution was observed in both MT2 and H9 cells. Given that A3G is restricted to the cytoplasm of cells [50], it is significant that the distribution of Peptide 2 was predominantly cytoplasmic as this suggests that disruption of Vif dimerization in the cytoplasm could have an effect on Vif interaction with A3G.

Recent studies have suggested that the major role for Vif in HIV-1 infectivity is to overcome the innate host defence of hA3G [36,37,43,51]. We therefore asked whether the antiviral effect of Peptide 2 was dependent on the expression of hA3G in the cells and viral Vif. HEK293T cells are permissive cells that do not naturally express hA3G however transfection with hA3G in these cells makes them nonpermissive to HIV-1 lacking Vif [15]. At OyaGen, Inc. pseudotyped HIV virions were produced in HEK293T cells by co-transfecting with HIV-1 proviral DNA (or ΔVif virus that is incapable of expressing Vif) and VSV-G with or without co-transfection with hA3G cDNA. Cells were either treated with PBS or Peptide 2.

**Figure 4:**
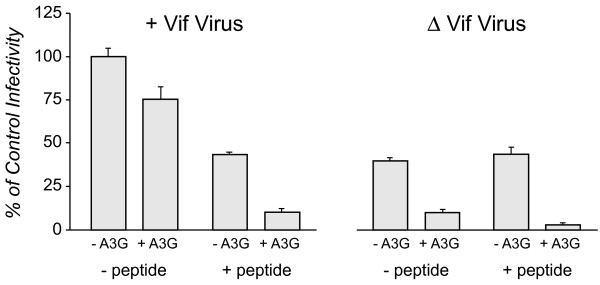
**Robust Antiretroviral Activity of Peptide 2 Requires Expression of Both Vif and A3G**. HEK293T cells were co-transfected with either VSV-G pseudotyped, + Vif (left panel) or ΔVif provirus (right panel), with or without A3G (as indicated below each histogram). During the incubation period, Peptide 2 was dosed into the specified samples (50 μM final concentration). The viral particles collected from the cell culture media over 48 h were normalized for their p24 content and incubated with JC53-bl cells for analysis of infectivity corresponding to luminescence as described in Methods. Infectivity of +Vif virions is shown as percent of the infectivity measured for +Vif/-hA3G/-peptide condition (14,498 red units). The (-) A3G/ΔVif virus control virions lacked Vif and the cells did not express A3G. Infectivity of the ΔVif virions is shown as percent of the infectivity measured with ΔVif/-hA3G/-peptide conditions (5,827 red units). The error bars represent the standard deviation with n = 3.

Viral particles released into the cell culture supernatant were harvested 24 h post transfection, normalized for p24 abundance and their infectivity quantified by luminescence using a HeLa cell system (JC53-bl) containing an LTR-driven luciferase reporter. Infectivity of +Vif virus produced under varying conditions is shown in the panel of histograms on the left of Figure 4. The infectivity of +Vif virus produced in the absence of hA3G and Peptide 2 (+Vif/-A3G/-peptide) was set to 100% for the purpose of this comparison. As expected, in the absence of peptide, the infectivity of +Vif virus produced in 293T cells expressing hA3G was not significantly different from the infectivity of +Vif virus in the absence of hA3G (left panel, first and second histograms) due to the ability of Vif to suppress the antiviral activity of hA3G. However, treatment of these producer cells with Peptide 2 significantly suppressed the infectivity of +Vif virus (left panel, third and fourth histograms, p ≤ 0.01, n = 3). Notably, the level of suppression of viral infectivity by the peptide was significantly greater (p ≤ 0.01, n = 3) when hA3G was expressed (left panel, compare the third and fourth histograms).

**Figure 5:**
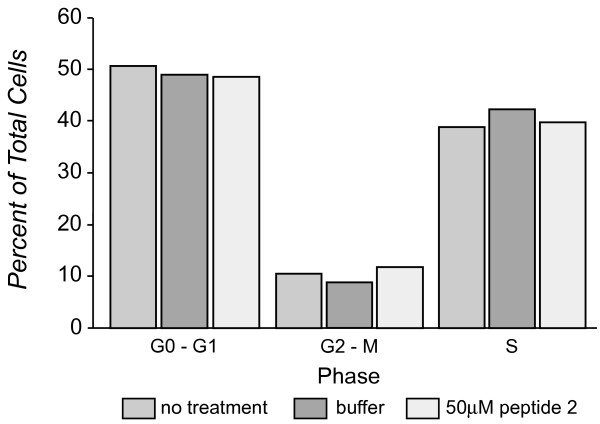
**Peptide 2 Does Not Affect Cell Cycle Progression**. Cultures of HEK293T cells at a starting confluency of 30% were either untreated, treated with buffer alone or with 50 μM Peptide 2 for 24 hours and processed for FACS analysis as described in Methods. The percent of cells in G1, S and G2/M phases of the cell cycle were calculated based on the DNA staining distributions.

While these data suggested a role for hA3G in the mechanism leading to the most robust antiviral activity of Peptide 2, it was surprising to find that the peptide also reduced viral infectivity of +Vif virus produced in cells lacking hA3G. This finding suggested that Vif multimerization supported viral infectivity through an hA3G-independent mechanism. To rule out non-specific effects, we evaluated whether peptide-treated cells had reduced viability or proliferation. Trypan blue exclusion analysis suggested that Peptide 2 only reduced cell viability by 6% compared to untreated cells over a 48 h period of dosing (data not shown). Cell cycle progression of untreated cells and cells treated with peptide for 24 h was evaluated by fluorescence activated cell sorting analysis of DNA content as described in Methods. The percent of the cell population in G1, S and G2/M phases of the cell cycle was similar under both conditions (Figure 5). Therefore reduced viral infectivity of the +Vif virus produced in peptide-treated cells lacking hA3G cannot be explained by off-target effects that altered cell viability or proliferation.

To demonstrate that Vif was required for the antiviral activity of Peptide 2 we evaluated the effect of peptide on ΔVif virus infectivity. The ΔVif produces fewer viral particles per ug of transfected plasmid than proviral DNA plasmids expressing Vif (see Figure 4 legend). Using p24 content to normalize virus input, the infectivity of ΔVif virus produced in the absence of hA3G and without Peptide 2 (ΔVif/-A3G/-peptide) was significantly reduced compared to +Vif virus in the absence of hA3G (set as the 100% infectivity control). In the absence of hA3G, ΔVif virion infectivity was not significantly affected by treating the producer cells with Peptide 2 (Figure 4, right panel, compare first and third histogram). As anticipated, hA3G expression in producer cells had a devastating effect on ΔVif viral infectivity, reducing viral infectivity to below ~10% (right panel, second histogram) of that seen with the +Vif virus minus hA3G. Treatment of the producer cells without hA3G with Peptide 2 appeared to further decrease the infectivity ΔVif virus (right panel, compare second and fourth histograms) however the difference in infectivity of ΔVif virus produced in untreated and treated cells is largely accounted for by the 6% reduced cell viability of peptide-treated cells as described above. We cannot rule out that the presence of Peptide 2 in these cells or possibly in the ΔVif viral particle may have had a deleterious effect on viral particles assembly or post entry replication. This is a possibility as the literature suggests that Vif itself may be assembled and processed in viral particles [52]. We conclude from our study that the most significant suppression of viral infectivity was observed when Vif and hA3G were co-expressed and that the efficacy of Peptide 2 is dependent on the expression of Vif.

At the time when Vif dimerization was described, it was not known that Vif prevented hA3G incorporation into viral particles and that Vif promoted hA3G ubiquitination and degradation and that [4-6,8-11,32,48,53]. We next asked whether treatment of cells with Peptide 2 would affect the recovery of hA3G with viral particles. OyaGen, Inc. produced pseudotyped HIV-1 virus particles in 293T cells co-transfected with hA3G cDNA with or without treatment with Peptide 2. Viral particles were harvested from cell culture media 24 h post-transfection and whole cell extracts were prepared. A representative number of cells (as whole cell extract) and a similar number of virions were resolved by SDS PAGE and western blotted from two separate experiments.

**Figure 6:**
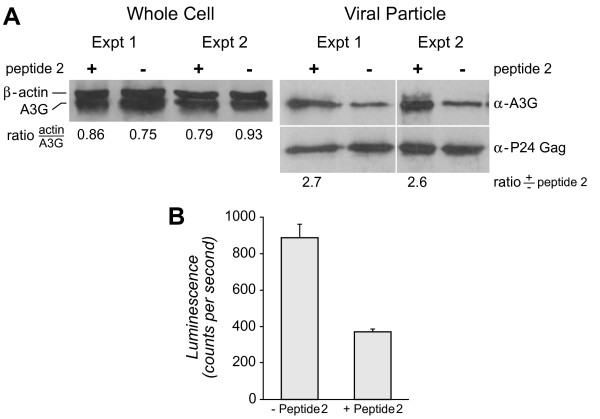
**Virions Treated with Peptide 2 Contain More A3G and have Reduced Infectivity**. (**A**) Virions collected from two separate experiments of HEK293T co-transfected with (+) Vif-provirus, VSV-G and A3G, with and without Peptide 2 treatments (50 μM), were normalized for p24 and sedimented through a sucrose cushion as described in Methods. The resultant pellets were lysed and resolved via SDS-PAGE and western blotted for A3G and p24. P24 was re-probed in the western blot in addition to the p24 ELISA quantification to validate the normalization. (**B**) Virion samples harvested from the co-transfection were normalized for p24 and infected into JC53-bl cells to quantify infectivity by luminescence analysis as described in Methods. Bars represent standard deviations with an n = 3.

Blots of whole cell extracts probed simultaneously with antibodies reactive with β actin and hA3G revealed that the expression of hA3G was similar in cells with or without peptide treatment (Figure 6A, left panel). Blots of viral particle proteins isolated from the cell culture supernatants were probed with antibody reactive with hA3G and then reprobed with antibody reactive with p24 (as a means of normalizing the recovery of hA3G with viral particles) (Figure 6A, right panel). These data demonstrated that virions released from cells treated with Peptide 2 had markedly greater recovery of hA3G relative to those released from cells that were not treated with the peptide. Analysis of the infectivity of p24-normalized virus demonstrated that viral particles prepared from cells treated with Peptide 2 had significantly (p < 0.01, n = 3) reduced infectivity (Figure 6B).

## Conclusion

We have addressed the therapeutic potential of the Vif dimerization domain as an antiretroviral drug target by providing the first independent confirmation that peptides, previously characterized as Vif dimerization antagonists, suppress HIV-1 infectivity. We have used a peptide mimetic of the Vif dimerization to confirm that the Vif dimerization interface is accessible in infected cells as a drug target and required for HIV-1 infectivity in nonpermissive cells. Co-expression of Vif and hA3G were necessary for a robust suppression of viral infectivity by the peptide. A novel finding in this study is that peptides previously shown to disrupt Vif dimerization enabled more hA3G to assemble with HIV-1 viral particles and enhanced the ability of hA3G to function as a post-entry host defence factor. The data explain why the most marked antiviral effect of the peptide was observed when Vif and hA3G were co-expressed. In fact, HIV-1 infectivity is strongly correlated with Vif-dependent reduction of hA3G assembly with viral particles [6,53]. The ability of Peptide 2 to reduce hA3G abundance in the viral particle without bringing about a reduction in total cellular hA3G supports literature suggesting that Vif, and in the case of our analysis, Vif multimers, may function to block hA3G assembly with virions through a mechanism that is separable from Vif-dependent hA3G degradation [51,54]. However, we hasten to add that hA3G was overexpressed in our system and is therefore higher in abundance than native expressed hA3G. Vif-dependent degradation of hA3G may not have been able to keep pace with the level to which hA3G was being overexpressed. If this was indeed the case, then it would leave open the possibility that Vif-dependent hA3G degradation may have taken place within a subcellular pool of hA3G that otherwise would have been directed into viral particle assembly pathway (such as newly translated hA3G). Peptide 2 acting as a Vif dimerization antagonist may have selectively affected the ability of Vif to block this pool of hA3G from assembling with viral particles.

We have also observed that Peptide 2 induced a reduction of +Vif virus infectivity in the absence of hA3G. This effect was not caused by reduced cell viability and proliferation due to peptide treatment. At face value the antiviral activity of the peptide in the absence of hA3G expression would suggest that Vif multimerization facilitates viral infectivity through a yet-to-be described mechanism. A related conclusion has been draw from other studies that found no evidence for overt changes in ΔVif virus viral replication or packaging in hA3G expressing cells and concluded that the defect in ΔVif virus replication was likely due to other functions of Vif [1].

The current leading hypothesis is that the primary role for Vif is to bind to hA3G and induced its degradation via the proteosome [4-11]. In this way, Vif prevents hA3G from being assembled with virions and acting as a post entry block to viral replication [6,12-15,17-21]. A role for Vif in viral infectivity other than to degraded hA3G is controversial. Examples of alterative functions for Vif include stabilization of reverse transcription complexes [47,55,56], efficient tRNALys/3 priming of reverse transcriptase complexes [57] and facilitating viral particle assembly [58-60]. Moreover, interactions between Vif and cellular proteins other than hA3G [61-63] and Vif phosphorylation by cellular kinases [64] have been reported as part of the infection process.

We cannot rule out that disruption of Vif multimers (i.e. the formation of Vif monomers) or the presence of Vif-Peptide 2 complexes could have impaired viral and host functions that otherwise would have supported viral infectivity. Moreover another area of controversy is whether Vif supports viral particle assembly and is packaged with virions [52,55,56,58-60]. Further studies will be necessary to determine whether Peptide 2 can be assembled with virions and exert its effect by inducing defects in viral particle assemble or post entry during viral replication.

In conclusion, the data present here suggested that dimers or higher order multimers of Vif were required for the interaction of Vif with hA3G and were an important part of the mechanism where by HIV-1 overcomes hA3G as an innate cellular defence factor. Validation of the Vif dimerization domain as an accessible target therefore holds promise for future therapeutic antiretroviral drug development.

## Methods

### Peptide design and synthesis

All peptides used in this study were synthesized by Davos Chemical Corp, Upper Saddle River, NJ or SigmaGenosys St. Louis, MO with > 95% purity. Peptides 1 and 2 were derived from sequence reported by Yang *et al*., from phage display peptides that disrupted Vif dimerization and blocked viral infectivity [29]. The control peptide was selected from human albumin sequence (accession # AAA98797) as a region of with no functional significance. HIV Tat sequence (YGRKKRRQRRRG) was included at the N-terminus of each peptide for cell transduction. For cell uptake studies, Peptide 2 was synthesized with a C-terminal FITC tag (Sigma Genosys). Cell cultures were dosed with the indicated final concentration of peptides from a 750 μM stock solution of peptide prepared fresh in phosphate buffered saline. Cell viability was assessed by a trypan blue exclusion assay preformed as described by the vendor (Invitrogen).

### Infectivity Assays and Quantification

Infectivity assays for Figure 1 were carried out as a fee for service by ImQuest BioSciences (Frederick, MD). For these studies MT-2 cells and the laboratory-adapted strain HIV-1_IIIB _were obtained from the NIAID AIDS Research and Reference Reagent Program, Rockville, Maryland. MT-2 cells were infected in 96-well microtiter plates at varying moi and cell density of 5.0 × 10^3 ^cells/well in a total volume of 200 μL. Infectivity was monitored by RT activity in each of the cultures at the indicated intervals. Peptides were added to the cultures on day one of the infection and every other day as the half-life of the peptide in media is 7–12 h (established by OyaGen, Inc., data not shown).

Viral replication was assessed at ImQuest BioSciences by quantifying reverse transcriptase activity in cell-free extracts. Reactions contained 1 mCi of 3H-TTP (1 Ci/mL, NEN) and poly rA and oligo dT at concentrations of 0.5 mg/mL and 1.7 Units/mL, respectively, from a stock solution which was kept at -20°C. For each reaction, 1 μL of TTP, 4 μL of dH_2_O, 2.5 μL of rAdT and 2.5 μL of reaction buffer were mixed. Ten microliters of this reaction mixture were placed in a round bottom microtiter plate and 15 μL of virus containing supernatant were added and mixed. The plate was incubated at 37°C in a humidified incubator and incubated for 90 min. Following reaction, 10 μL of the reaction volume were spotted onto a DEAE filter, washed 5 times for 5 min each in a 5% sodium phosphate buffer, 2 times for 1 min each in distilled water, 2 times for 1 min each in 70% ethanol, and then air dried. The dried filter was subjected to scintillation counting in Opti-Fluor O.

Pseudotyped HIV production for infectivity assays and viral particle production were carried out by OyaGen, Inc. HEK293T cells passaged into 6-well plates were co-transfected 0.5 μg pDHIV3-GFP and 0.5 μg VSV-G, courtesy of Dr. Baek Kim (Department of Microbiology, University of Rochester, NY), and 1.0 μg A3G expressing plasmid courtesy of Dr. Harold Smith's laboratory using FuGENE 6 Transfection Reagent (Roche, Indianapolis, IN). The cells were dosed 4 h and 8 h after transfection with Peptide 2 to bring a final concentration of 50 μM, assuming that all of the peptide was consumed at the time of the each dosing. 24 h after transfection, the media was replaced with fresh media containing 50 μM Peptide 2. 24 and 48 h after transfection, the media was passed through a 0.45 micron SFCA syringe filter and analyzed for viral particle density via p24 ELISA (Zeptometrix) and read in a Wallac 1420 plate reader (Perkin Elmer, Watham, MA). The data for infectivity were evaluated by a two tailed probability analysis.

### Fluorescence Activated Cell Sorting

Cultures of HEK 293T cells at 50% confluency were dosed with buffer or Peptide-2 as described above and fixed in 70% ethanol (4°C) for 12 h. Cells were resuspended to 0.3 × 10^3 ^cells/ml in PBS and RNA digested with 1 mg/ml RNase A (Sigma) at 37°C for 30 min. Cells were brought to 20 ug/ml propidium iodide and filtered through 37 um mesh. Fluorescence activated cell sorting was performed by the University of Rochester Cell Sorting core facility as a fee for service.

### Western Blotting Viral Particles for A3G

Fifteen ng p24-equivalent viral particles were pelleted through 2 mL 20% sucrose solution in PBS at 148,000 × g for 2 h. The supernatant was drawn off and the viral particles were resuspended in 50 μL lysing buffer composed of 1× Reporter Lysis Buffer (Promega) and 1 pellet/10 mL lysing solution containing Complete^® ^EDTA-free protease inhibitor (Roche). The viral particle lysates were processed three times by freezing to -20°C, thawing in a 37°C water bath and vortexing for 10 seconds. The lysates were acetone precipitated and re-pelleted at 15,000 × g before aspirating and resuspending in SDS PAGE sample buffer. The lysates were resolved via 10.5% SDS-PAGE and transferred to BioTrace^®^NT nitrocellulose membrane (Pall, West Chester, PA) and probed for A3G with rabbit anti-A3G primary antibody #10084, (NIH AIDS Research and Reference Resource Program), and goat anti-rabbit peroxidase conjugated secondary antibody (Invitrogen, Carlsbad, CA). To verify the p24 normalization determined in the ELISA, the membranes were probed with mouse anti-p24 primary antibody #3537 (NIH AIDS Research and Reference Resource Program) and goat anti-mouse peroxidase conjugated secondary antibody (Kirkegaard & Perry Laboratories, Gaithersburg, MD). Following the secondary antibodies, the membranes are incubated with Western Lightning Chemiluminescence Reagent Plus (Perkin Elmer) and recorded on X-OMAT film (Kodak, Rochester, NY). The resultant bands were quantified using NIH ImageJ 1.36b software.

### Viral Particle Infectivity Assay

JC53-bl cells (NIH AIDS Research and Reference Resource Program) were passaged by OyaGen, Inc into 96-well plates at 10,000 cells/well, 75 μL volumes. The viral particles were diluted to 6000 pg p24/mL and added to triplicate wells, 25 μg volumes, to the cells when they appeared 40–50% confluent. 48 h after infection, 100 μL of Steady Glo Reagent (Promega, Madison. WI) was added to each well and allowed to incubate for 7 minutes at room temperature before reading the luminescence in a Wallac 1420 Multilabel Counter (Perkin Elmer).

### Fluorescence microscopy

OyaGen, Inc treated MT2 and H9 cells in culture with 50 μM of Peptide 2-FITC (Sigma, MO) for varying durations and then centrifuged onto glass slides. The cells were fixed with 2% paraformaldehyde in PBS for 5 min at 4 oC and permeabilized with 0.4% Triton X 100 (Sigma) for 5 min at 4 oC and washed extensively in PBS. Cells were mounted in DAPI-containing media (Vectasheild, Vector Labs, Burlingame, CA) and viewed by with an Olympus BH-2 fluorescence microscope (Orangeburg, NY) and photographed through with an 8.0 megapixel Olympus SP-350 camera equipped with an eyepiece telescope.

## Competing interests

OyaGen, Inc is privately held HIV/AIDS biotech start-up company focusing on the development of therapeutics based on the APOBEC family of proteins. OyaGen holds a world-wide exclusive license on the United States Patent Application 10/688,100 granted from the Thomas Jefferson University, Philadelphia, PA entitled "US PCT 10/688,100 "Multimerization of HIV-1 VIF Protein as a Therapeutic Target", filed by Drs. Hui Zhang, Roger J. Pomerantz and Bin Yang and Thomas Jefferson University.

HCS is the founder and chief scientific officer of OyaGen, Inc, and principle shareholder. His salary is supported through NIH extramural support and the University of Rochester. He directed the research in this paper and wrote the article as part of his paid consultant time with OyaGen, Inc.

## Authors' contributions

JHM is a full time technical associate employed by OyaGen, Inc. and has no equity staked in the company. He carried out the majority of the research and participated in the writing of the manuscript.

VP was a summer intern and University of Rochester undergraduate who participated in carrying out the experiments on cell uptake of peptide and fluorescence microscopy.

HCS is the founder and Chief Scientific Officer of OyaGen, Inc., Rochester NY. He is the principle equity holder in the company and serves as CSO of OyaGen as a paid consultant. He is a tenured full professor in the Department of Biochemistry and Biophysics at the University of Rochester, Rochester, NY. HCS designed the experiments, analyzed the data and wrote the manuscript.
